# Assessing the Utility of Photoswitchable Fluorescent Proteins for Tracking Intercellular Protein Movement in the *Arabidopsis* Root

**DOI:** 10.1371/journal.pone.0027536

**Published:** 2011-11-23

**Authors:** Shuang Wu, Koji Koizumi, Aurora MacRae-Crerar, Kimberly L. Gallagher

**Affiliations:** Department of Biology, University of Pennsylvania, Philadelphia, Pennsylvania, United States of America; Instituto de Biología Molecular y Celular de Plantas, Spain

## Abstract

One way in which cells communicate is through the direct transfer of proteins. In plants, many of these proteins are transcription factors, which are made by one cell type and traffic into another. In order to understand how this movement occurs and its role in development, we would like to track this movement in live, intact plants in real-time. Here we examine the utility of the photoconvertible proteins, Dendra2 and (to a lesser extent) EosFP as tags for studying intracellular and intercellular protein movement in the *Arabidopsis* root. To this end, we made fusions between Dendra2 and six mobile transcription factors. Our results show that Dendra2 is an effective tool for studying protein movement between plant cells. Interestingly, we found that Dendra2 could not simply be swapped into existing constructs that had originally contained GFP. Most of the fusions made in this way failed to produce a fluorescent fusion. In addition we found that the optimal settings for photoconversion of Dendra2 in stably transformed roots were different from what has been published for photoconversion in transient assays in plants or in animal cells. By modifying the confocal setting, we were able to photoconvert Dendra2 in all cell layers in the root. However the efficiency of photoconversion was affected by the position of the cell layer within the root, with more internal tissues requiring more energy. By examining the Dendra2 fusions, we confirmed the mobility of the SHORT-ROOT (SHR) and CAPRICE (CPC) transcription factors between cells and we further discovered that SHR movement in stele and CPC movement in the epidermis are non-directional.

## Introduction

In both plants and animals, proteins traffic between cells. Many of these proteins serve as intercellular signals that provide positional information during the development of the organism [1]–[3]. Others convey metabolic status or transduce signals from the environment [4], [5]. To study the functions of these molecules, one would like to track their movement *in vivo*. One way in which this has been done (particularly in plants) is through the microinjection of fluorescently labeled proteins or the co-microinjection of a protein along with fluorescent dextrans into single cells within an organ. (e.g. the tobacco leaf) [6]. These experiments provided much information about protein movement both within and between cells and tissues. However, they are limited to regions of the organism that are accessible to microinjection or to studying the protein outside of its native tissue. In addition, microinjection is an intrusive process that by its nature wounds the cell. As cell-wounding responses have the potential to affect intercellular trafficking [7], less disruptive means to examine protein movement are desirable.

**Figure 1:**
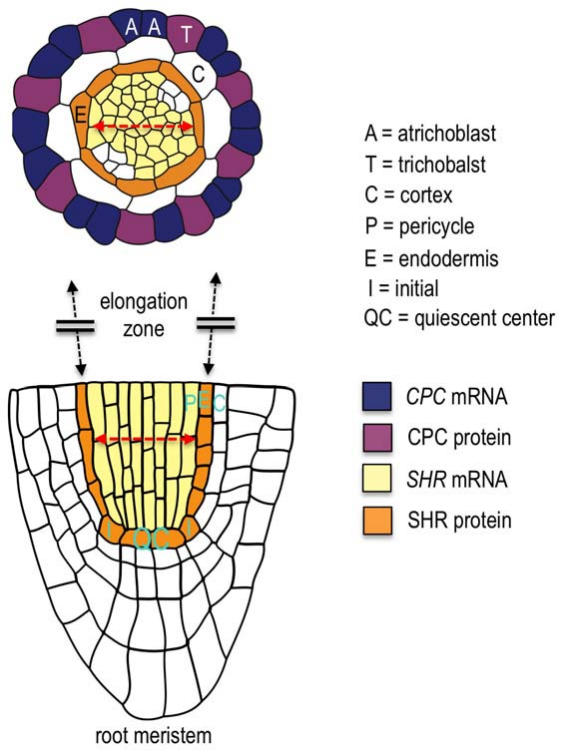
Diagram of the *Arabidopsis* root. (A) A color-coded tracing of a transverse cross section above the root meristem. (B) Longitudinal cross section through the root meristem. All relevant tissues and cell types are labeled. The extent of the stele tissue (the tissue internal to the endodermis) is indicated by the double red arrows. The expression patterns of two of the mobile proteins examined in this study, SHR and CPC are shown. The CPC protein moves from atrichoblasts (non-hair cells) into the trichoblast (hair cells); the SHR protein moves from the stele to the endodermis.

The discovery and cloning of GFP (Green Fluorescent Protein) revolutionized the study of protein movement [8]. It is now relatively easy in most systems to fuse a protein of interest to GFP and examine protein localization in stably transformed tissues. GFP and other genetically encoded fluorescent proteins have also made FRAP (fluorescent recovery after photobleaching) experiments possible in live cells. GFP tagged proteins can be bleached in the cell or domain of interest and then recovery monitored to detect protein movement. However, using FRAP, it is not always possible to determine the direction of protein movement, or to examine protein movement in the domain in which the protein is expressed. For example, the SHORT-ROOT (SHR) protein, which is the focus of many of our experiments, is expressed in the *Arabidopsis* root throughout most of the stele (Figure 1). The SHR protein moves out from the stele into the neighboring endodermis, QC and initial cells [9], [10]. SHR movement from the stele into the neighboring endodermal cells is observed using FRAP; however FRAP assays are not helpful in looking at movement within the stele, because protein movement is indistinguishable from synthesis of new SHR protein.

In principle, protein movement could be examined using an inducible promoter or a cell specific promoter with expression that is restricted to a smaller subset of cells than the endogenous expression domain. In practice, however, it is still difficult to achieve cellular specificity using an inducible promoter system and there can be a significant lag time between induction and appreciable gene expression [11]. Cell specific promoters are preferable, but the cells and tissue for which these are available are still limited. Therefore, the precise analysis of protein dynamics and movement would be advanced by tools that specifically highlight sub-populations of the target proteins to study their behavior in the context of the entire domain of protein expression.

Recently, photoconvertible proteins were introduced to the field of live cell imaging. These proteins can be irreversibly “switched” or “converted” from one color to another (often from green to red) in response to a specific wavelength of light [12]. In their native states, many of these proteins like Kikume [13], EosFP [14], and Kaede [15] oligomerize making them problematic for use as tags to study protein movement. In animal systems, researchers have made good use of modified forms of EosFP (mEosFP and tdEosFP) and Dendra (Dendra2) that behave as monomers [14], [16], [17].

Despite their successful use in animals, there have been relatively few reports of their use in plants. Outside of transient expression assays in tobacco [18] and *Arabidopsis leaves*, there is a report by Dhonukshe *et al.* in which they made stably transformed plants expressing PIN2-EosFP. There are also exciting reports by Mathur *et al.* in which they used mEosFP to mark various endomembrane compartments, organelles and the cytoskeleton [19], [20]. Rausin *et al.* made fusions of RSZp22 to Dendra2 and was able to express this protein transiently in tobacco, but was not able to use this construct to make stably transformed *Arabiodpsis* seedlings [21]. To our knowledge there are no reports for the use of Dendra2 as a fusion protein in stably transformed *Arabidopsis*. Here we report the cloning and characterization of protein fusions to Dendra2 (hereafter D2), tdEosFP, and mEosFP that are stably expressed in *Arabidopsis*. We focused our analysis on six root expressed non-cell autonomous transcription factors that had previously been characterized as GFP fusion proteins and that represent six different diverse protein families (Table S1) [10], [22], [23]. In particular we concentrated on the examination of SHR movement as we had previously characterized SHR movement using structure-function analysis and shown that movement is targeted [9], [22], [24]. Likewise the movement of CPC is also regulated and specific (Figure 1) [23].

From these experiments we found that D2, mEosFP and tdEosFP are not strictly interchangeable with GFP with respect to localization and function of the protein being interrogated. Fusions of tdEosFP with SHR affected both the subcellular localization and intercellular movement of the SHR protein. We also found that the conditions for photoconversion of D2 in the intact root differed from those reported previously for animal cells [16]. We report our settings using the Zeiss 710 as a starting point for further analysis. As the actual laser power that impinges upon the plane of focus (even on the same model confocal) can vary based upon the age and alignment of the laser, the type of objectives used the thickness of the sample and the light transmission characteristics of the cells, the setting provided in the paper are intended as a starting point for analysis and to demonstrate how individual settings can be manipulated to achieve photoconversion, while reducing photobleaching.

By examining different protein fusions, we found that we could achieve photoconversion of D2 in all cell files in the root. The efficiency of photoconversion correlated with the location of target cells within the tissue with more internal tissues requiring greater laser power. Examination of fusions of SHR to D2 confirmed the movement of SHR from the stele into endodermis. In addition we show that SHR can move between stele cells and that CPC movement between cells in the epidermis is non-directional Our results suggest that D2 is a valuable tool for studying protein movement in intact root cells, however there are limitations to its usefulness. It may not function well as a tag with all proteins and is not optimal for the study of rapid processes within the cell.

## Results and Discussion

The *Arabidopsis* root has well defined cell files, is optically clear and relatively thin with a diameter of 120–150 µm. Under the correct conditions, roots can survive for many hours, even days, on a standard microscope slide with cover glass making it an excellent system for *in-vivo* analysis of protein movement. To better understand how proteins move among cells in the root, we examined six different mobile proteins, each representing a different family of transcription factor (Table 1; Table S1). We fused all of these proteins to D2 (and in some cases mEosFP or tdEosFP; Table 1) and assayed for fluorescence, convertibility and movement.

**Table 1:** Summary of the stably transformed lines made for this study.

Construct ID	Fluorescence
*p35S:Dendra*	**Y** (38/56)
*p35S:At4g37650-Dendra2 (SHR)*	**N** (0/48)
*p35S:At2g46410-Dendra2 (CPC)*	**Y** (28/40)*
*p35S:At4g00940-Dendra2*	**Y** (19/28)
*p35S:At2g22850-Dendra2*	**N** (0/6)
*p35S:At4g27410-Dendra2*	**N** (0/6)
*p35S:At4g37940-Dendra2*	**N** (0/5)
*pSHR:At4g37650-Dendra2 (SHR)*	**N** * (1/176)
*pSHR:At4g37650-tdEosFP (SHR)*	**Y** (2/5)
*pSHR:At4g37650-mEosFP (SHR)*	**N** (0/5)
*pCPC:At2g46410-mEosFP (CPC)*	**N** (0/22)
*pSHR:At4g37650-NL-Dendra2 (SHR)*	**Y** (6/20)

Y = Yes, N = No fluorescence. The numbers in parenthesis indicates the number of fluorescent lines over the total made.

*Only one line had fluorescence.

### Creation of photoconvertible fusion proteins

**Figure 2:**
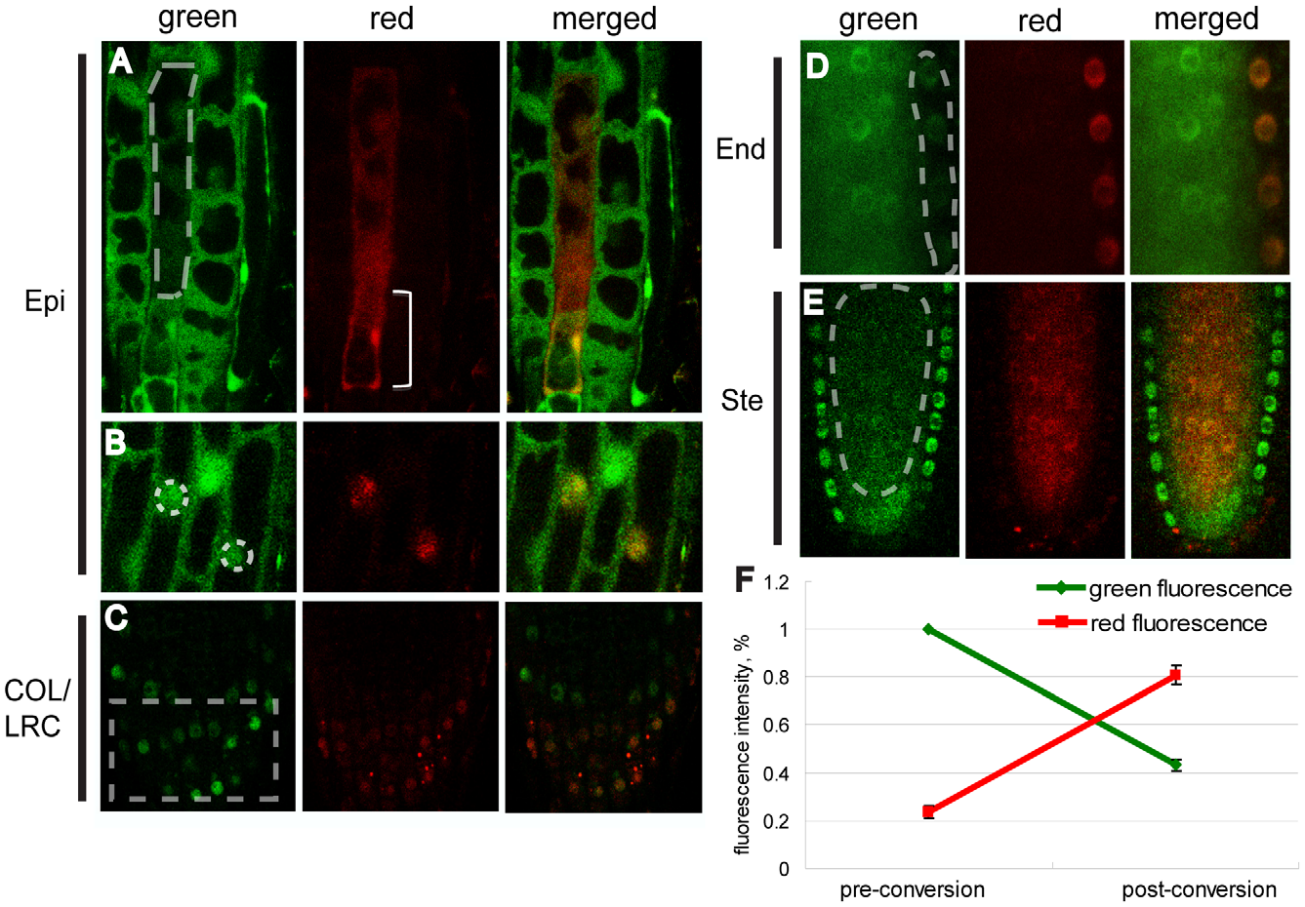
Green-to-red photoconversion of D2 in different cell types in the *Arabidopsis* root. Dotted lines indicate the region of interest (ROI) for photoconversion. All images presented were collected immediately after the photoconversion. (A) Free D2 was converted in the cytoplasm of an expanded epidermal (Epi) cell. By the time the conversion was achieved, a population of the converted protein had moved into the lower portion of this cell (bracket). (B) CPC-D2 was converted in the nuclei of two epidermal cells. (C) Dof-D2 was converted in multiple cells of the columella root cap and lateral root cap (COL/LRC). SHR-NL-D2 was converted (D) in the endodermis (End) and (E) stele (Ste) cells. (F) The relative fluorescence of SHR-NL-D2 in the endodermis in the red channel increases to approximately 60% of the pre-conversion green fluorescence, while the green fluorescence drops to around 40% of the pre-conversion level (sixty iterations with 10% laser power).

The proteins shown in Table 1 were previously described as amino-terminal fusions to GFP that showed fluorescence in the roots of stably transformed *Arabidopsis* seedlings [10], [22], [23]. As all of the fusions shown in Table 1 were made using the Gateway 3-way recombination system (Invitrogen), we used the same strategy (and the same cDNA clones kindly provided by Ji-Young Lee) to make fusions to D2 (Dendra2-At; Evrogen). The full-length constructs were placed under the control of the *35S* promoter [25] and transformed into wild-type plants. The roots of T2 (the second generation following transformation) seedlings were examined. Only two of the six proteins made with this cloning strategy showed appreciable fluorescence (Table 1 and Figure 2A–C). Both CPC-D2 and At4g00940-D2 (hereafter Dof-D2) proteins were expressed throughout all cells in the root. The Dof-D2 protein localized to nuclei (Figure 2C); whereas CPC-D2 (Figure 2B) showed both nuclear and cytoplasmic localization, which is similar to CPC-GFP (Figure S1A and B) [23]. Consistent with the function of CPC, all CPC-D2 seedlings with fluorescence lacked epidermal trichomes and produced an excessive number of root hairs, indicating that the fusion protein is functional [26]–[29]. The function of the Dof protein has not been characterized. Roots expressing Dof-D2 were not obviously different from wildtype.

The *p35S* fusions of D2 to SHR, At2g22850, At4g27410 and At4g37940 showed no fluorescence (Table 1). Since silencing of the *35S* promoter is not uncommon, we expressed *SHR-D2* from the *SHR* promoter. Of the 176 individual T2 lines that we examined, only 1 showed very weak fluorescence (Table 1). Deuschle *et al.* reported ubiquitous transgene silencing (i.e. none of the transformants showed fluorescence) when trying to stably express genetically encoded glucose sensors in *Arabidopsis* that had been used successfully in mammalian cells [30]. To overcome this problem, they transformed the sensors into the *rdr6* mutant background, which is deficient in transgene and trans-acting siRNA silencing [30]. To test whether the lack of fluorescence in our case was due to transgene induced gene silencing, we crossed the *pSHR:SHR-D2, p35:SHR-D2*, *p35:At2g22850-D2*, *p35:At4g27410-D2* or *p35:At4g37940-D2* constructs into the *rdr6* background and examined the F2 seedlings. In addition we directly transformed the *pSHR:SHR-D2* construct into the *rdr6* background and examined the T2s. None of the above fusion proteins showed fluorescence in the *rdr6* mutants, suggesting that the lack of fluorescence is primarily the result of improper protein folding and/or protein instability and not transgene silencing.

In an effort to create a photoconvertible form of SHR, we switched our focus to the two monomeric forms of EosFP (mEosFP and tdEosFP) [14], [17], [31] and made an alternative version of D2 with a flexible amino acid linker at the amino teminus. Neither *pSHR:SHR-mEosFP* nor a control construct that we made with *CPC (pCPC:CPC-mEosFP)* showed fluorescence in the root. In contrast, the *pSHR:SHR-tdEosFP* showed very bright fluorescence, but did not show the normal cytoplasmic and nuclear localization in the stele that is seen with the SHR-GFP protein (Figure S1) [9], [10], [24]. In addition, the SHR-tdEosFP was not present in the endodermis (Figure S1E, G and H), suggesting that the tdEosFP fusion blocks SHR movement into the endodermis.

In the transient expression assays in tobacco leaves reported by Martin, they used amino acid linkers of various composition and length between their proteins of interest and the photoconvertible tag [18] presumably to promote proper folding. To test if the addition of a linker would make a difference with the *SHR-D2* fusions, we constructed a modified version of *D2* that contains DNA sequences that code for the amino acid sequence: NAAIRS at the amino terminus. This sequence was chosen because Wilson *et al.* had shown it to be particularly flexible in its ability to fold [32]. The *pSHR:SHR-NAAIRS-D2* (NL-D2) resulted in a fluorescently tagged SHR protein. SHR-NL-D2 localized to the nucleus and cytoplasm of the stele cells and was detected in the nuclei of the endodermis indicating that SHR-NL-D2 maintains SHR mobility (Figure 2 D, E and Figure S1C and for comparison D).

### Photoconversion of Dendra2

As a starting point to determine the appropriate conditions for conversion of D2 using a laser scanning confocal, we examined roots expressing *pSHR:SHR-NL-D2* and applied the settings published previously for use in animal cells [16]. After illumination of SHR-NL-D2 expressing samples with the 405 nm laser, we detected a significant decrease in the green signal but no noticeable red fluorescence, suggesting that under these conditions we bleached the fluorophore. To achieve photoconversion and avoid excessive bleaching, we modified the original confocal settings. In the end we were able to convert free D2 and the D2 fusion proteins in all cell types in the root (Figure 2A–E). For example, when we used 10% laser power with sixty iterations, SHR-D2 in the endodermis showed an approximately 60% decrease in green fluorescence, with a concomitant increase in red fluorescence (Figure 2D, E). After correcting for background autofluorescence in the root, the intensity of converted red fluorescence was approximately 60% of the pre-converted green signal (Figure 2F).

### Factors affecting photoconversion

**Figure 3:**
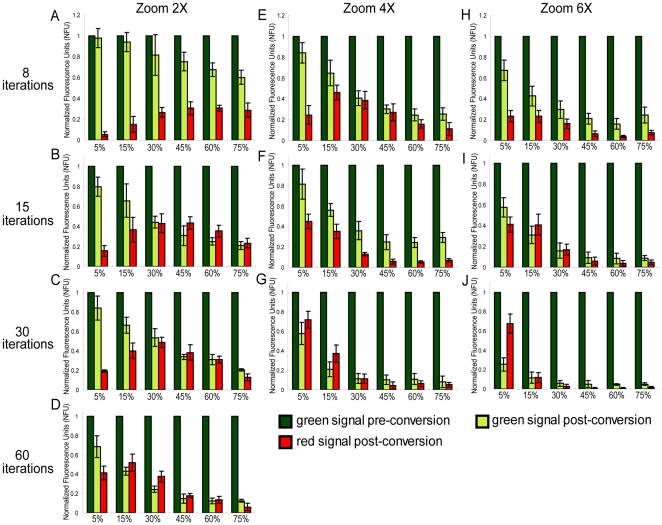
The laser power, number of iterations, and zoom settings affect the effectiveness of photoconversion of SHR-D2 in stele cells. Increased laser powers (5%, 15%, 30%, 45%, 60%, and 75% as indicated) were examined for conversion efficiency combined with different numbers of iterations. (A, E and H) eight iterations; (B, F and I) fifteen Iterations; (C, G and J) thirty iterations; (D) sixty iterations and zoom settings (2×, 4×, and 6× as indicated). The fluorescence intensity of both post-conversion green signal and red signal are normalized to pre-conversion green signal
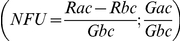
 before setting the Y-axis to 1.0 (see materials and methods: R_bc_ = red levels before conversion and R_ac_ = red levels after conversion; G_bc_ = green levels before conversion and G_ac_ = green levels after conversion). The optimal conversion (strong increase in the red signal and minimal bleaching of the green) at a zoom setting of 2× was achieved with 15–30% laser power and 30 iterations. A further increase in the number to sixty caused a significant increase in photobleaching. Note that 6× approximates the calculated zoom for optimal sampling density.

One of the attractions to using a photoconvertible protein as a marker for protein movement is that the converted protein can be monitored within the context of the total protein population (i.e. both the unconverted and the converted proteins can be monitored within the same cell or domain). Therefore it is advantageous to maximize photoconversion, while minimizing photobleaching. In order to determine the optimal conditions for achieving this in the *Arabidopsis* root, we incrementally increased laser power and tried different numbers of iterations. Using SHR-NL-D2 in the stele as an example, we found that laser powers below 5% were insufficient for noticeable photoconversion. In contrast laser powers above 30% resulted in significant bleaching. Good results were achieved for SHR-NL-D2 in stele cells using 30% of full laser power with thirty iterations; the post-conversion green signal decreased to approximately 55% of the pre-conversion green fluorescence, and the red signal produced was equivalent to approximately 50% of the pre-conversion green fluorescence (Figure 3A–D).

A drawback of using repeated iterations to achieve photoconversion is that it can take up to 8 sec (with 30 iterations). If the process being examined occurs quickly, this may be prohibitively long. For example in Figure 2A, in the time that it took to achieve photoconversion of free D2 within the cytoplasm of this epidermal cell, the protein had already spread throughout the rest of the cell and into the nucleus. To decrease the period of conversion, fewer iterations with higher laser intensity, or dwell time can be used. In some cases we were able to reduce the number of iterations by directly increasing the dwell time. However, we found the results to be variable, and the risk of bleaching to be much higher with increased dwell time than with increased iterations (data not shown).

Another way to increase the laser dwell time is to adjust the zoom factor. Increasing the zoom factor reduces the region of the specimen that is scanned and simultaneously increases the duration that the laser dwells on each individual point per line. When imaging live tissues, it is often advantageous to adjust the zoom settings so that only the region of interest is sampled and to take advantage of the full resolution of the objective being used. To empirically determine the conversion settings for different zooms, we examined the photoconversion efficiency of *pSHR:SHR-D2* in the stele. When the zoom was set to 4×, which allows the observation of the whole root meristem and part of the root expansion zone, 15% laser power with only 8 iterations gave similar results to 30% power with 30 iterations (at 2× zoom; Figure 3C), but reduced the time of photoconversion by more than half. 5% laser power with thirty iterations gave the highest level of photoconversion relative to photobleaching. Similar levels of red signal were achieved using the same condition with a zoom of 6×, but there was also a significant decrease in the green signal compared to 4× zoom (Figure 3E–J).

To determine what effect the position of the tissue within the organ has on photoconversion, we looked at CPC-D2 in root epidermal cells. With 30 iterations, using only 5% laser power we achieved levels of CPC-D2 photoconversion similar to those observed when using 25% laser power and 30 iterations on SHR-D2 in stele cells. Use of laser powers greater than 30% caused considerable bleaching of CPC-D2 in epidermal cells (Figure S2). To determine whether the tissue or the identity of the D2 fusion affected the photoconversion, we examined free D2 in the epidermis and stele and found that they were nearly identical to the results achieved with CPC-D2 and SHR-D2 respectively (Figure S3). These results show that as imaged cells are farther from the surface of the organ, increased laser powers are required for phtotoconversion of D2. However, we can not entirely rule out some influence of the fusion partner on the efficiency of photoconversion as the energy required for conversion Dof-D2 in the epidermis was higher than for CPC and free D2 (data not shown). Therefore, the parameters for photoconversion may differ slightly for different proteins even in the same tissues. However in all cases the optimal settings for photoconversion in the root differed significantly from those provided by the supplier of D2 for photoconversion of the protein in COS cells using the Zeiss 710 (100% laser power with a dwell time of 10 µsec and five iterations; www.evrogen.com)

### D2 fusion proteins are mobile

**Figure 4:**
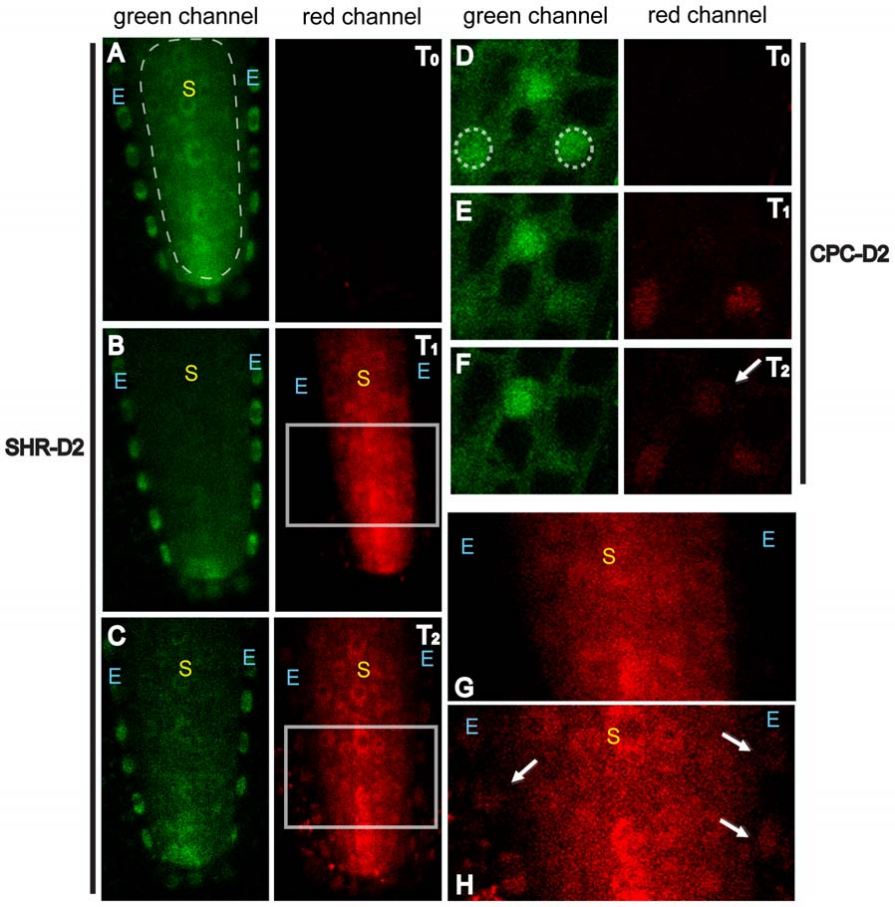
The converted forms of SHR-NL-D2 and CPC-D2 can move in the Arabidopsis root. (A–C, G, H) SHR-NL-D2 in the stele “S” and endodermis “E.” (D–F) CPC-D2 in the epidermis. The ROI for conversion is indicated by dotted lines (A) and (D). T_0_ is pre-conversion; T_1_ is immediately following photoconversion and T_2_ is 2.0 hr after conversion in (C) and 20 min in (F). The region within the square in (B) is magnified in (G) and the region in (C) is magnified in (H). In all panels arrows point to cells into which the converted proteins have moved.

Intercellular movement of transcription factors is a critical signaling mechanism during root development. In the root meristem, the SHR protein moves from stele into the neighboring cells where it promotes the formation of the endodermis [10]. In the epidermis the CPC protein is made in the non-hair cells and moves into the incipient hair cells where it promotes hair cell fate [23]. As the SHR-NL-D2 construct is expressed from the SHR promoter the presence of fluorescence in both the stele and the endodermis (Figure 4A) indicated that the fusion protein in its native (unconverted) state is mobile. To test whether the converted (red) forms of SHR-NL-D2 and CPC-D2 are also mobile, we observed the converted SHR-NL-D2 and CPC-D2 in endodermal and epidermal cells respectively. We found that when converted in the stele and monitored over time, the red form of SHR-NL-D2 can be detected in the endodermis (Figure 4G and H, 2.0 hr post-conversion) indicating that the converted form is also mobile. The time frame for movement of SHR-NL-D2 was consistent with FRAP results using SHR-GFP, in which approximately 50% recovery of fluorescence was achieved after 2.0 hr [33].

To examine movement of CPC-D2, we converted CPC-D2 specifically in the nuclei of epidermal cells. We found that CPC-D2 could move between all epidermal cells (Figure 4F, 20 min post-conversion). These results show that fusion of D2 to CPC does not block its ability to move between cells. Furthermore we found that CPC-D2 moves isodiametrically between cells in the epidermis (i.e. movement was not specifically from non-hair to hair cells). The lack of apparent preference for the direction of CPC movement between root epidermal cells is in contrast to movement of CPC and the related protein, ENHANCER of TRY and CP3 (ETC3) in trichome patterning where bi-directional movement seems to be inhibited by binding of these proteins to GLABRA3 (GL3), which apparently traps the protein in the nucleus. These results suggest that nuclear trapping may not regulate CPC movement in the root.

**Figure 5:**
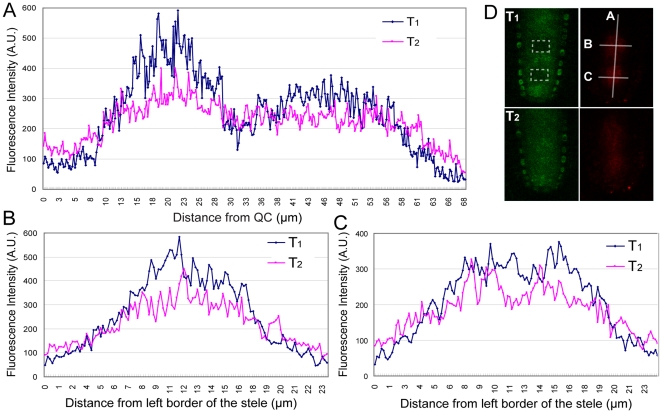
SHR-NL-D2 is able to move within the stele. Photoconversion of SHR-D2 was performed in two regions within the stele (dotted lines in D) and then the appearance of the red form was monitored and plotted in the correspondingly labeled graphs along the A, B and C axes, which are drawn in panel (D). T_1_ is immediately after photoconversion and T_2_ is 90 min after photoconversion. The plotted values of fluorescence intensity are an average of six to nine measurements along the indicated axis using 1.5 µm intervals. The t-test value for the plotted data in (A) is 4.1125E-17 for region 0–12 µm along x-axis; 3.78806E-07 for 29 µm–39 µm along x-axis and 7.35549E-09 for 58 µm–68 µm along x-axis. The t-test value for the plotted data is 1.70681E-06 in (B) and 2.71241E-07 in (C).

Using traditional FRAP, we can detect the movement of SHR from stele cells into the endodermis. But with FRAP, it is difficult to determine whether SHR movement occurs throughout the stele. To examine this, we converted small regions within the meristem of the stele tissue. We found that movement of SHR-D2 occurred in both the transverse and longitudinal directions, suggesting that SHR can move between cells within the stele (Figure 5). Fusion of SHR to tdEosFP expressed from the SHR promoter was restricted to the stele suggesting that SHR-tdEosFP is immobile. To test whether the SHR-tdEosFP could move within the stele we converted the protein in a subset of stele cells and then assayed for movement. We were unable to detect the converted SHR-tdEosFP protein outside of the region of photoconversion indicating that the fusion protein is not mobile (Figure S1 E–H). In the stele the SHR-tdEosFP fusion did not localize properly and appeared to form small aggregates within these cells, indicating a problem with protein folding. These results suggest that SHR movement is not directed specifically towards the endodermis, instead other mechanisms must account for SHR's specific accumulation in the endodermis. Previous papers have implicated SCARECROW (SCR) and JACKDAW (JKD) in trapping SHR in the nuclei of endodermal cells [34], [35]. Non-directional movement followed by nuclear trapping therefore may account for the high levels of SHR in the endodermis.

In summary, photoconvertible proteins are useful tools for studying protein movement in stably transformed *Arabidopsis* roots. Labeling of SHR and CPC with D2 did not affect protein mobility or function and we were able to detect movement of both CPC and SHR in living cells. However we found there to be much trial-and-error involved in choosing the appropriate tags for fusion. These results suggest that D2, mEosFP and tdEosFP are not as amenable as GFP to use as a fluorescent marker. In addition, attention must be paid to the function of the protein of interest, as the presence of fluorescence did not necessarily indicate functionality. The optimal settings for photoconversion in the root were different than what has been previously published in animal cells and were affected by the position of the cells within the tissue being examined. We found that optimal photoconversion of Dendra2 fusion proteins could be reliably achieved with minimal photobleaching by combining low laser power with multiple iterations. As a general principle, when first attempting photoconversion of D2 we recommend starting with a low laser power (5–10%) and 15–30 iterations to avoid photobleaching. Both the laser power and the number of iterations can be adjusted based on the effieciency of photoconversion and the amount of photobleaching for each individual protein. If higher zooms are applied, both the laser power and the number of iterations should be decreased to avoid photobleaching. If utilized properly, D2 can be a powerful tool for studying protein dynamics, movement and stability. However, since the photoconversion process in the root was significantly slower than what has been shown in animals, D2 may not be ideal for monitoring processes that occur quickly in the root.

## Materials and Methods

*tdEosFP* was amplified from a pcDNA3-F1-EosFP vector [36] by PCR with primer sets: B2FEosFP 5′GGGGACAGCTTTCTTGTACAAAGTGGGCATGAGT GCGATTAAGCCAGAC and B3REosFP 5′GGGGACAACTTTGTATAATAAAGTT GCTTATCGTCTGGCATTGTCAGG. *Dendra2-At* amplified from the Dendra2-At-NT vector (Evrogen) using primers: B2FDendra GGGGACAGCTTTCTTGTACA AAGTG GGCATGAACACTCCTGGAATCAATC and B3RDendra 5′GGGGACAAC TTTGTATAATAAAGTTGCTCATTTGTACACACCTGAGTCTCC. For construction of NAAIRS-Dendra2 (NL-D2) 18 bps of sequence was added in the forward primer B2FNAAIRSDen2 5′GGGGACAGCTTTCTTGTACAAAGTGGGCAACGCTGCTA TCAGATCTATGAACACTCCTGGAATCAATC. This sequence was designed to conform to codon usage bias in *Arabidopsis thaliana*. The *EosFP* and the *Dendra* constructs were recombined into pDONRP2R-P3 using standard protocols of Gateway BP reaction (Invitrogen). The *mEosFP* sequence was amplified from the *tdEosFP* construct introducing the published V123T and T158R substitutions [14] using standard molecular biology techniques. After verifying the sequences, these plasmids were used for 3-way recombination reaction with the *35S*, *SHR* and/or *CPC* promoters all in the pDONRP4-P1R plasmid [22], along with the full-length cDNAs in pDONR221 and the dpGreen BarT destination vector [22] (standard Gateway protocols; Invitrogen). The resulting binary vectors were introduced into Agrobacterium strain GV3101-pSoup-pMP and transformed into *Arabidopsis* Col-0 plants.

### Plant Materials and Growth Conditions

Stable transgenic plants were generated using the standard floral dip transformation method. For selection of T1 seedlings on soil: upon germination seedlings were sprayed 3 times/week with 340 µl/l Finale solution (BACKED by BAYER). For selection of T1 seedlings on plates: seeds were grown on 0.5× MS medium (Caisson) containing 0.05% (w/v) MES (pH 5.7), 10 mg/ml Gulfosinate-ammonium (Sigma), and 0.5% (w/v) Phytagel (Sigma) in a growth chamber at 23°C under 16 hr light/8 hr dark cycle. For analysis of fluorescence, all seeds were sterilized using 70% household bleach (6.15% sodium hypochlorite; Clorox) and then plated on 0.5× MS medium (Caisson) containing 1% (w/c) granulated agar (BBL) and 1% (w/v) sucrose. The seeds were incubated vertically for 5–6 days at 23°C with 16 hr of light per day to allow the seeds to germinate and produce roots.

### Microscopy and Photoconversion

*Arabidopsis* seedlings with intact roots were placed on slides in a drop of water for short-term imaging or in liquid MS medium (same as above without agar) for extended observations. Confocal analysis was conducted on a Zeiss LSM 710 laser scanning confocal microscope using a Zeiss LD C-Apochromat 40×/1.1 NA water immersion objective lens (Carl Zeiss Microimaging Inc.). On the Ziess LSM 710 confocal, we used the bleaching mode with ZEN 2009 software (Carl Zeiss Microimaging Inc.) with various attenuations of the 405 nm laser power and iterations (which is defined as one complete pass with the 405 nm laser over the region being scanned) to convert the indicated proteins. The pixel dwelling time for photoconversion was set to 1 µsec. The green fluorescence of D2 was observed using 10% power of the 488 nm laser with 900 V master gain; while red fluorescence was observed using 20% power of the 561 nm laser with 900 V master gain. All images were captured in 512×512 formats (equivalent to 106.1 µm×106.1 µm at 2× zoom-in; 53 µm×53 µm at 4× zoom-in and 35.4 µm×35.4 µm at 6× zoom-in). During the time-course observation, the first image after photoconversion was taken immediately after the “bleach series” and the following images were taken 20 min to 2 hr later.

One way to provide the guidelines for successful photoconversion is to report the actual energy required for photoconversion. However, it is difficult to determine the precise energy involved in photoconversion based upon only the confocal settings as after the light passes through the fiber optic cables and the objective the actual energy reaching the sample is much lower than the original laser output. When we measured actual laser power using a power meter, use of 20% power (from the 30 mW 405 nm laser) sends 0.22 mW to the sample; 10% power sends 0.13 mW and 5% sends 0.07 mW. Ideally, the diameter of the focused laser beam using the Zeiss LD C-Apochromat 40×/1.1 NA water immersion is 450 nm [1.22×(405/1.1)]. Therefore, a rough estimation of the energy perceived by the sample using 10% laser power is approximately 0.34 mW/µm^2^. Taken into consideration the pixel dwell time of 1.0 µsec, each µm^2^ would receive around 7.6×10^−7^ J/µm^2^ when the iterations used is sixty and the zoom is 2. This estimation is higher than previously reported in animal cells in which conversion was achieved using a 405 nm laser. The difference between our conditions and those previously published however is that they used a parked laser beam with continuous irradiation to a fixed point. Compared to those recommended by Evrogen, our settings deliver much less energy to the cells. Using the setting recommended by Evrogen (for use in animal cells), for CPC-D2 we would considerably bleach the fluorophore.

### Data processing

To evaluate the levels of photoconversion, the fluorescence intensity of both green and red versions of the fluorophore were measured using Image-J software. The absolute increase in red fluorescence (iRF) was measured as: R_bc_- R_ac_ , where R_bc_ = red levels before conversion and R_ac_ = red levels after conversion. Likewise the decrease in green fluorescence (dGF) was calculated as: G_bc_- G_ac_ where G_bc_ = green levels before conversion and G_ac_ = green levels after conversion. In order to be able to compare the iRF to the dGF both of these values were normalized to (divided by) G_bc_ to get the normalized fluorescence units (NFU). These values are presented in the bar graphs in Figures 3, 4, S2 and S3. To show the actual direct increase of red signal, background was not subtracted from post-conversion red signal in Figure 2F. Instead, both pre-conversion background and post conversion red signal were normalized to the preconversion green signal as a percentage.

## Supporting Information

Figure S1 **A comparison of different fluorophores.** (A) CPC-D2 and for comparison (B) CPC-GFP. (C) SHR-NL-D2 and for comparison (D) SHR-GFP. (E–H) SHR-tdEosFP in the stele. Note the abnormal localization in stele cells and absence of signal in endodermis. Although the protein is not mobile, SHR-tdEosFP can be converted on the confocal. (E) and (H) the green signal prior to and after conversion respectively. (F) Signal in the red channel prior to and (H) after conversion. “E” = endodermis.
(TIF)

Figure S2 **Photoconversion of CPC-D2.** Conversion of CPC-D2 in epidermis using three different laser powers (as shown) and 30 iterations. 40% laser power caused considerable bleaching of both the green and red signals.
(TIF)

Figure S3 **The cell type or position of the cell with the tissue affects the amount of laser power required for photoconversion of D2.** (A) Photoconversion of D2 was performed with 5% laser power combined with 30 iterations. The fluorescence intensity of both post-conversion green signal and red signal are normalized to preconversion green signal. All images were obtained at a 2× zoom (512×512 pixels and 106.1 µm×106.1 µm). In the epidermis, both CPC-D2 and free D2 showed similar conversion efficiency. However using the same conditions, both SHR-D2 and free D2 in stele cells showed lower conversion efficiency than CPC-D2 or free D2 in the epidermis.
(TIF)

Table S1 **Expression pattern and movement of the mobile transcription factors used in this study.** A = atrichoblast; B = procambium; C = cortex; CEI = cortical/endodermal initials; D = Epidermis; E = Endodermis; L = Columella; M = phloem; P = pericycle (pp = phloem pole; xp = xylem pole); QC = quiescent center; T = tricoblast; X = xylem.
(DOCX)
