# Supplementary figures and images for: Assessing the Utility of Photoswitchable Fluorescent Proteins for Tracking Intercellular Protein Movement in the Arabidopsis Root
Source: PLoS One. 2011 Nov 23;6(11):e27536. doi: 10.1371/journal.pone.0027536 (PMC3223184; doi:10.1371/journal.pone.0027536)

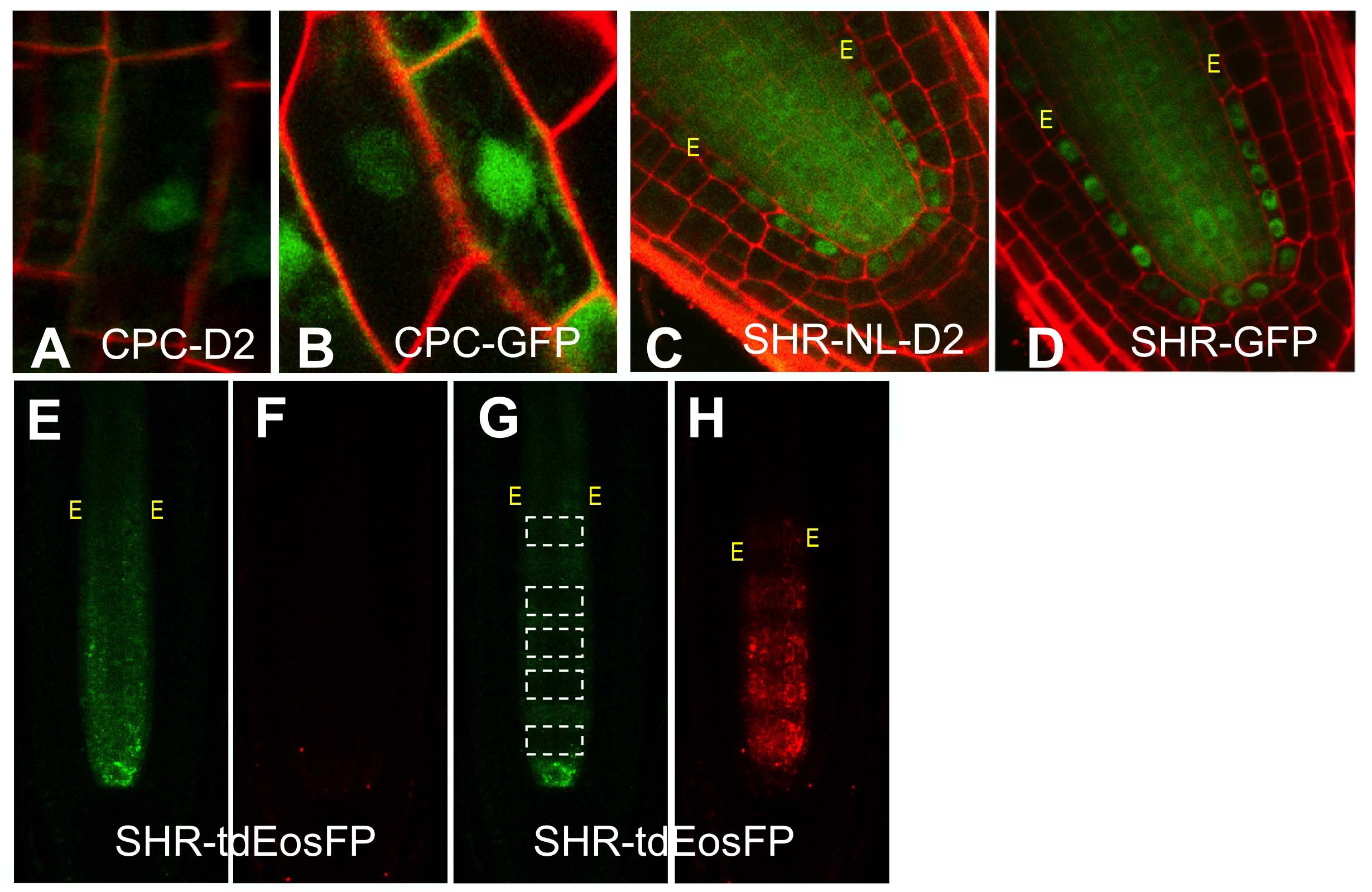

Supplement: Figure S1 — A comparison of different fluorophores. (A) CPC-D2 and for comparison (B) CPC-GFP. (C) SHR-NL-D2 and for comparison (D) SHR-GFP. (E–H) SHR-tdEosFP in the stele. Note the abnormal localization in stele cells and absence of signal in endodermis. Although the protein is not mobile, SHR-tdEosFP can be converted on the confocal. (E) and (H) the green signal prior to and after conversion respectively. (F) Signal in the red channel prior to and (H) after conversion. “E” = endodermis. (TIF) [file pone.0027536.s001.tif]

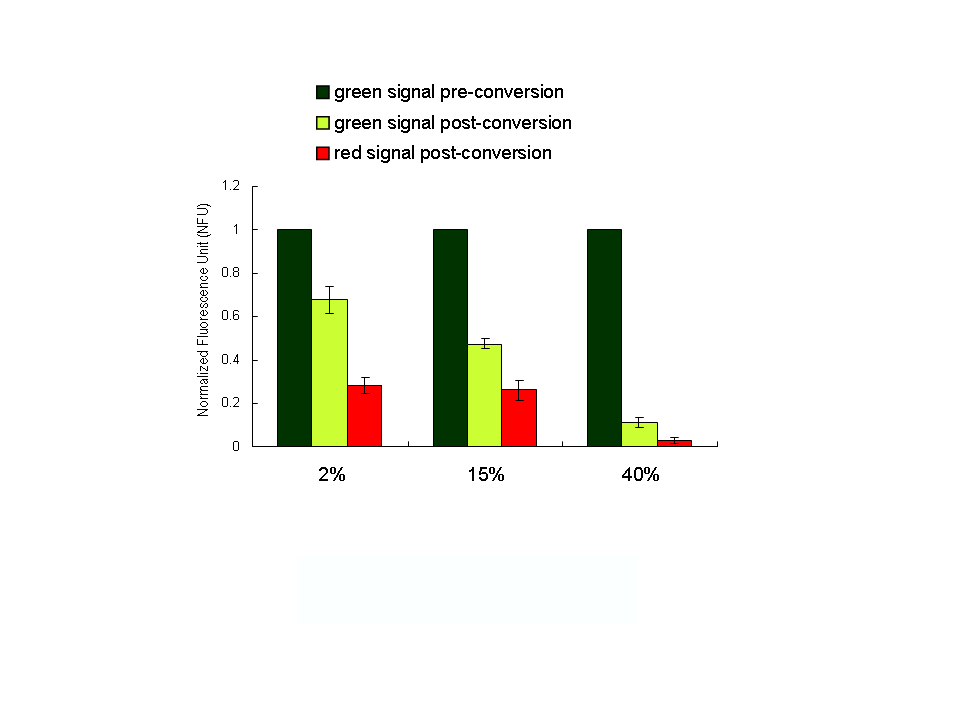

Supplement: Figure S2 — Photoconversion of CPC-D2. Conversion of CPC-D2 in epidermis using three different laser powers (as shown) and 30 iterations. 40% laser power caused considerable bleaching of both the green and red signals. (TIF) [file pone.0027536.s002.tif]

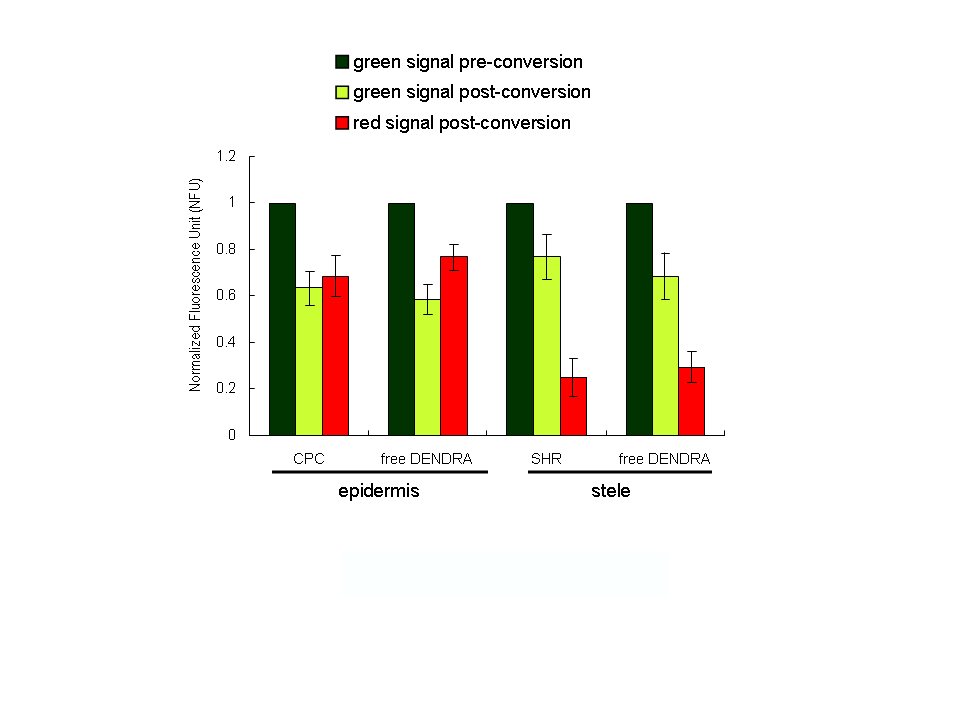

Supplement: Figure S3 — The cell type or position of the cell with the tissue affects the amount of laser power required for photoconversion of D2. (A) Photoconversion of D2 was performed with 5% laser power combined with 30 iterations. The fluorescence intensity of both post-conversion green signal and red signal are normalized to preconversion green signal. All images were obtained at a 2× zoom (512×512 pixels and 106.1 µm×106.1 µm). In the epidermis, both CPC-D2 and free D2 showed similar conversion efficiency. However using the same conditions, both SHR-D2 and free D2 in stele cells showed lower conversion efficiency than CPC-D2 or free D2 in the epidermis. (TIF) [file pone.0027536.s003.tif]
