# Supplementary material for: Assessing the Utility of Photoswitchable Fluorescent Proteins for Tracking Intercellular Protein Movement in the Arabidopsis Root
Source: PLoS One. 2011 Nov 23;6(11):e27536. doi: 10.1371/journal.pone.0027536 (PMC3223184; doi:10.1371/journal.pone.0027536)
Supplement: Table S1 — Expression pattern and movement of the mobile transcription factors used in this study. A = atrichoblast; B = procambium; C = cortex; CEI = cortical/endodermal initials; D = Epidermis; E = Endodermis; L = Columella; M = phloem; P = pericycle (pp = phloem pole; xp = xylem pole); QC = quiescent center; T = tricoblast; X = xylem. (DOCX) [file pone.0027536.s004.docx]

| **Gene ID/Protein FaMily** | **Moves From** | **Moves To** |
| --- | --- | --- |
| At4g37650 (SHR) /**GRAS** | B, X, P(xp) | E, M, P(pp), QC, CEI |
| AT2G46410 (CPC) /**Myb** | A | T |
| At4g00940 /**C2H2** **Dof** | P | P and E |
| At2g22850 /**bZIP** | P(pp) | P(xp) and E |
| At4g27410 /**NAM** | P, E and D | C and D |
| At4g37940 /**MADS-Box** | L, B | All Cells |
| **Supplemental Table 1. Expression pattern and movement of the mobile transcription factors used in this study**. A=atrichoblast; B=procambium; C=cortex; CEI=cortical/ endodermal initials; D=Epidermis; E=Endodermis;; L=Columella; M=phloem; P=pericycle (pp=phloem pole; xp=xylem pole); QC=quiescent center; T=tricoblast; X=xylem | | |
